# Diagnostic and prognostic benefit of arterial spin labeling in subacute stroke

**DOI:** 10.1002/brb3.1271

**Published:** 2019-03-25

**Authors:** Thoralf Thamm, Sarah Zweynert, Sophie K. Piper, Vince I. Madai, Michelle Livne, Steve Z. Martin, Cornelius X. Herzig, Matthias A. Mutke, Eberhard Siebert, Thomas Liebig, Jan Sobesky

**Affiliations:** ^1^ Center for Stroke Research Berlin (CSB) Charité ‐ Universitätsmedizin Berlin, corporate member of Freie Universität Berlin, Humboldt‐Universität zu Berlin, and Berlin Institute of Health Berlin Germany; ^2^ Department of Neurology Charité ‐ Universitätsmedizin Berlin, corporate member of Freie Universität Berlin, Humboldt‐Universität zu Berlin, and Berlin Institute of Health Berlin Germany; ^3^ Institute of Biometry and Clinical Epidemiology Charité ‐ Universitätsmedizin Berlin, corporate member of Freie Universität Berlin, Humboldt‐Universität zu Berlin, and Berlin Institute of Health Berlin Germany; ^4^ Berlin Institute of Health (BIH) Berlin Germany; ^5^ Department of Neurosurgery Charité ‐ Universitätsmedizin Berlin, corporate member of Freie Universität Berlin, Humboldt‐Universität zu Berlin, and Berlin Institute of Health Berlin Germany; ^6^ Department of Neuroradiology Heidelberg University Hospital Heidelberg Germany; ^7^ Department of Neuroradiology Charité ‐ Universitätsmedizin Berlin, corporate member of Freie Universität Berlin, Humboldt‐Universität zu Berlin, and Berlin Institute of Health Berlin Germany; ^8^ Department of Neuroradiology Ludwig‐Maximilian‐University Munich Germany; ^9^ Department of Neurology Johanna‐Etienne‐Hospital Neuss Germany

**Keywords:** arterial spin labeling, cerebral perfusion, ischemic stroke, outcome prediction

## Abstract

**Background and Purpose:**

Brain perfusion measurement in the subacute phase of stroke may support therapeutic decisions. We evaluated whether arterial spin labeling (ASL), a noninvasive perfusion imaging technique based on magnetic resonance imaging (MRI), adds diagnostic and prognostic benefit to diffusion‐weighted imaging (DWI) in subacute stroke.

**Methods:**

In a single‐center imaging study, patients with DWI lesion(s) in the middle cerebral artery (MCA) territory were included. Onset to imaging time was ≤7 days and imaging included ASL and DWI sequences. Qualitative (standardized visual analysis) and quantitative perfusion analyses (region of interest analysis) were performed. Dichotomized early outcome (modified Rankin Scale [mRS] 0–2 vs. 3–6) was analyzed in two logistic regression models. Model 1 included DWI lesion volume, age, vascular pathology, admission NIHSS, and acute stroke treatment as covariates. Model 2 added the ASL‐based perfusion pattern to Model 1. Receiver‐operating‐characteristic (ROC) and area‐under‐the‐curve (AUC) were calculated for both models to assess their predictive power. The likelihood‐ratio‐test compared both models.

**Results:**

Thirty‐eight patients were included (median age 70 years, admission NIHSS 4, onset to imaging time 67 hr, discharge mRS 2). Qualitative perfusion analysis yielded additional diagnostic information in 84% of the patients. In the quantitative analysis, AUC for outcome prediction was 0.88 (95% CI 0.77–0.99) for Model 1 and 0.97 (95% CI 0.91–1.00) for Model 2. Inclusion of perfusion data significantly improved performance and outcome prediction (*p* = 0.002) of stroke imaging.

**Conclusions:**

In patients with subacute stroke, our study showed that adding perfusion imaging to structural imaging and clinical data significantly improved outcome prediction. This highlights the usefulness of ASL and noninvasive perfusion biomarkers in stroke diagnosis and management.

## 1 INTRODUCTION

Predictors of clinical outcome after stroke are necessary to identify high‐risk patients and to guide therapy. Current stroke imaging protocols including diffusion‐weighted imaging (DWI) are robust predictors of stroke outcome (Lutsep et al., 1997; Sorensen et al., 1996) but do not inform on cerebral hemodynamic alterations.

Arterial spin labeling (ASL) is a noninvasive magnetic resonance imaging (MRI) method to measure brain perfusion. Unlike O‐15 water positron emission tomography (PET) or dynamic susceptibility contrast (DSC) MRI, it allows estimation of cerebral blood flow (CBF) without radiation exposure or contrast agent application.

In 2014, the ISMRM Perfusion Study Group recommended the implementation of ASL into standard stroke imaging protocols (Alsop et al., 2015). Given the fast evolvement, the limited generalizability, and the heterogeneity in sequences and parameters, clinical experience with ASL is still limited and needs further clinical validation.

Methodological studies reported a moderate to good correlation between ASL and DSC or PET for CBF measurement in chronic and acute cerebrovascular disease (Martin et al., 2015; Mutke, 2014; Nael et al., 2013; Golen, 2014; Wang et al., 2012, 2013; Zhang, 2014). In acute stroke (<24 hr after onset), perfusion asymmetries detected by continuous ASL perfusion MRI (CASL‐PI) correlated well with symptom severity and outcome after 3 months (Chalela et al., 2000). Furthermore, transient arterial artifacts of the occluded vessel were described as predictors of early reperfusion during thrombolysis (Okazaki et al., 2016) and early ASL hyperperfusion was associated with favorable outcomes after thrombolysis (Kohno, Okada, Yamagata, Takayoshi, & Yamaguchi, 2016; Viallon et al., 2010), while also indicating a higher risk of hemorrhagic transformation (Yu et al., 2015).

Previous reports focus on ASL imaging during the hyperacute and acute phase of stroke to guide recanalization therapy (Bivard, Stanwell, Levi, & Parsons, 2013; Yu, 2017). As the first days following acute stroke are vulnerable and may be crucial for patient outcome, stroke units were established to prevent or rapidly treat complications after stroke (Anonymous, 1997; Cavallini, Micieli, Marcheselli, & Quaglini, 2003; Fuentes & Diez‐Tejedor, 2009). Brain perfusion is highly dynamic within the first days and some patients may show mismatch patterns beyond 24 hr after stroke (Gonzalez, 2010). This underlines the need for extended hemodynamic validation in order to guide interventions for, for example, blood pressure management or further interventions in patients with high hemodynamic risk.

In this study, we aimed to evaluate the performance of a certified and commercially available ASL imaging sequence in the subacute phase of stroke as a perfusion biomarker to predict early clinical outcome.

## 2 METHODS

### 2.1 Patients

Consecutive patients admitted to our stroke unit were included into this study according to the following criteria: (a) unilateral DWI lesion(s) in the middle cerebral artery (MCA) territory, (b) onset to imaging time ≤ 7 days, and (c) MRI with readable ASL‐CBF and DWI. Clinical parameters were collected from the electronic medical records. The study was approved by our institution's ethics committee.

### 2.2 Image acquisition and imaging parameter

The standard clinical stroke protocol was performed on Siemens Magnetom MRI scanner systems: Either 1.5 (Aera) or 3 Tesla (Skyra) (Siemens Healthcare, Erlangen, Germany). DWI was performed with a b‐value of 1,000 s/mm^2^, repetition time (TR) 10,300 ms, echo time (TE) 98 ms, voxel size 0.6 × 0.6 × 3.0 mm^3^, slice thickness 3.0 mm, and acquisition time 2:36 min. A 3D time‐of‐flight (TOF) intracranial MR angiography (MRA) was performed using the following parameters in four 3D‐blocks: TR 21 ms, TE 3.43 ms, voxel size 0.3 × 0.3 × 0.5 mm^3^, and acquisition time 4:45 min.

The ASL product sequence included a multi‐inversion time (TI) pulsed ASL (PASL) with the following details: flow alternating inversion recovery (FAIR) labeling using a Q2TIPS saturation scheme, anterior‐posterior labeling, six postlabeling delay (PLD) time points between 600 ms and 3,600 ms, TR: 4,800 ms, TE: 36.52 ms, bolus: 700 ms, background suppression, two repetitions. The image readout was performed using single‐shot 3D‐gradient and spin echo (GRASE) echo‐planar imaging (EPI) with the following parameters: voxel size 4.0 × 4.0 × 5.0 mm^3^, slice thickness of 5.0 mm. These parameters were chosen to allow a clinically relevant acquisition time of 2 min. The certified commercial sequence did not provide the option of M0 acquisition for absolute quantification.

### 2.3 Data processing and analysis

Images were postprocessed and analyzed offline. MeVisLab (MeVis Medical Solutions AG, Bremen, Germany) and FSL (Oxford Centre for Functional MRI of the Brain (FMRIB), UK) were used for multi‐TI processing and calculating cerebral brain perfusion (CBF) and bolus arrival time (BAT) maps with assumed arterial blood water T1 of 1.66 s and 1.3 s for the 3.0 T and 1.5 T studies, respectively (Alsop et al., 2015; Lu, Clingman, Golay, & van Zijl, 2004). ITK‐SNAP (University of Pennsylvania, Philadelphia, PA, USA) was used for segmentation and Vinci (Max‐Planck‐Institute, Cologne, Germany) for co‐registration and viewing.

For the qualitative analysis, two board‐certified expert raters, a neuroradiologist (E.S.) and a neurologist (J.S.), reviewed the obtained imaging data visually. Raters had access to DWI and information on the vessel status (TOF‐MRA and extra‐/transcranial duplex ultrasound) but were blinded to clinical status and outcome. The review comprised of an evaluation of image quality (1: very good, 2: good, 3: sufficient, and 4: uninterpretable), asymmetry, and the presence of visually conspicuous perfusion patterns (decreased/increased perfusion, arterial transit delay artifact [ATDA]) in both ASL‐CBF and ASL‐BAT maps. DWI lesions were classified as small (lesion diameter ≤1.5 cm), medium, or large (encompassing the entire distribution area of the MCA) as reported previously (Kohno et al., 2016). In cases of divergent ratings, the two raters reached a consensus during the same session.

For the quantitative analysis, relative signal intensities for DWI and ASL‐CBF maps were calculated. Since the commercially available ASL sequence on site did not measure a M0, the quantification of cerebral perfusion was not feasible. The quantitative analysis thus determined relative signal intensities and no absolute values of cerebral perfusion. To calculate the relative signal intensities, the infarct core on DWI maps and the perfusion alteration on ASL‐CBF maps were outlined manually on every affected slice, resulting in volumes of interest (VOI). The intensity of perfusion alteration within the VOI was described by the mean voxel value and normalized to a manually selected region of interest (ROI) comprising the contralateral MCA territory (relative CBF, rCBF). This ROI was outlined on an axial image at the level of basal ganglia and contained cortical and juxtacortical areas within the MCA territory.

To identify relevant perfusion alterations, study data were defined as follows:

- Relative CBF (rCBF) = mean ASL‐CBF VOI/mean ASL‐CBF contralateral MCA ROI
- Hypoperfusion = rCBF ≤70% and/or presence of ATDA
- Hyperperfusion = rCBF ≥130%

Thresholds for relevant perfusion alterations (Ezura, Takahashi, & Yoshimoto, 1996) stratified into the following subgroups: (a) no or minor perfusion alteration (normal), (b) hypoperfusion and/or ATDA, and (c) hyperperfusion. Furthermore, patient records were screened for National Institutes of Health Stroke Scale (NIHSS) scores (admission and discharge), modified Rankin Scale (mRS) score (discharge), and TOF‐MRA and duplex ultrasound findings. A stenosis was considered severe if its degree was ≥70% (NASCET criteria) in duplex ultrasound (Arning, 2010).

### 2.4 Statistical analysis

Normally distributed variables are reported as mean and standard deviation (*SD*) whereas quantitatively skewed variables and scores are reported as median and the limits of the interquartile range (IQR) given as 25th and 75th percentile.

Chi‐squared test (Fisher's exact or Pearson's) was applied to compare frequencies between groups. Nonparametric tests included Mann–Whitney *U* test or Kruskal–Wallis test, for comparing metric or ordinal variables between two or more independent groups, respectively. Statistical significance was indicated by a significance level below 5%. No adjustment for multiple comparison was done. All test results constitute exploratory data analysis.

### 2.5 Regression model

Two binary logistic regression models with dichotomized outcome (mRS 0–2 vs. 3–6) as dependent variable were calculated. Model 1 (DWI) contained logarithmic DWI lesion volume as the independent variable, as well as clinical and demographic traits as covariates (age, admission NIHSS, ipsilateral severe stenosis or occlusion of internal cerebral artery (ICA) or middle cerebral artery (MCA), and acute therapy (thrombolysis and/or endovascular treatment)). Model 2 (DWI + ASL) additionally contained a compound parameter for ASL‐CBF perfusion pattern. This parameter consisted of the following two dimensions: type of altered ASL perfusion (symmetry, hypoperfusion, or hyperperfusion) and relevance (relative CBF [rCBF] ≤70%, or ≥130%, or ATDA). The area‐under‐the‐curve (AUC) of the receiver‐operating‐characteristic (ROC) analysis was calculated and reported with 95% confidence interval (CI) for each model. The likelihood‐ratio‐test was used for the comparison of both models by testing whether the goodness of fit significantly changed when ASL perfusion pattern is added (Seshan, Gonen, & Begg, 2013).

Statistical analyses were performed using SPSS (version 24.0, SPSS Inc., Chicago, IL, USA) and Stata (version 14.0, StataCorp LLC, College Station, TX, USA).

## 3 RESULTS

### 3.1 Patient characteristics

Between May 2015 and October 2016, 38 patients met the inclusion criteria. The median age was 70, admission NIHSS 4, and discharge mRS 2. The median time between symptom onset and imaging was 67 hr. In 22 patients (58%) imaging was performed using a 3 T scanner. DWI lesions were small (median 1.3 ml [IQR 0.4–9.9 ml]), of lacunar origin in 53%, and had strategic stroke localization in 21% (defined as thalamic or capsular involvement). Eleven patients (29%) had received intravenous thrombolysis and four patients (11%) additionally underwent endovascular recanalization prior to MRI. Thirteen patients (34%) had a severe stenosis or occlusion of the middle cerebral artery (MCA) or internal carotid artery (ICA).

**Figure 1:**
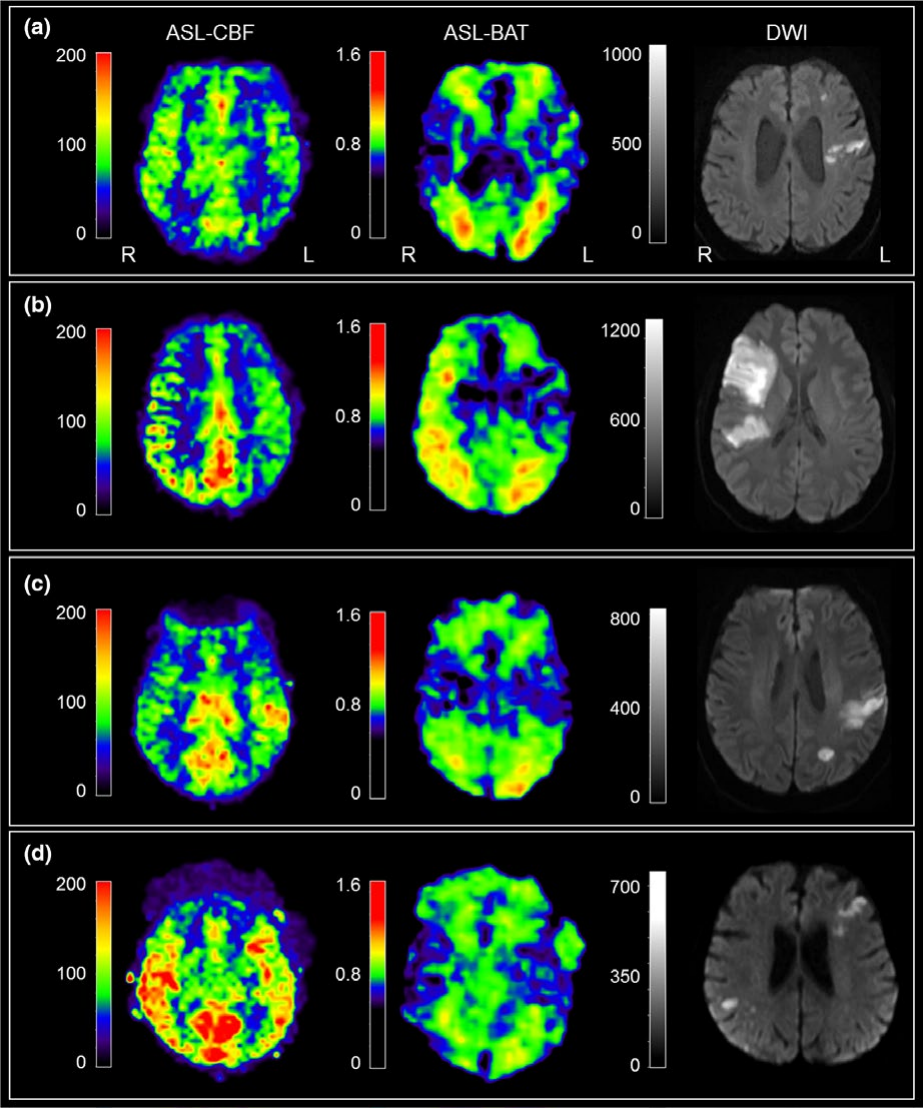
Representative ASL‐CBF and ASL‐BAT maps, corresponding DWI, and a summary of symptoms are displayed. Colorbars for ASL‐CBF and ASL‐BAT maps show signal intensities, not absolute perfusion values. (a) Scattered DWI lesions and a hemispheric hypoperfusion without ASL‐BAT alterations. A 75‐year‐old‐male patient presenting with right‐sided hemiparesis and aphasia, NIHSS of 8 at admission, treated with intravenous thrombolysis. MRI (3T) after 49 hours revealed a hypoperfusion within the left MCA territory: rCBF = 66%. Patient was discharged with NIHSS = 1 and mRS = 2. (b) Large DWI lesion with an ipsilateral delay in bolus arrival (ASL‐BAT map), a hypoperfusion, and an ATDA in the ASL‐CBF map. A 38‐year‐old‐female patient presenting with a left‐sided sensorimotor hemisyndrome due to a dissection and occlusion of the right carotid artery. NIHSS of 4 at admission. MRI (3T) after 4 days shows hypoperfusion and delayed blood flow (ATDA) in the affected right MCA territory. rCBF = n/a due to ATDA. Patient was discharged with NIHSS = 4 and mRS = 2. (c) DWI lesion within the posterior MCA territory with a corresponding ASL‐CBF hyperperfusion and no ASL‐BAT alterations. A 56‐year‐old‐male patient presenting with right‐sided hemiparesis and aphasia, NIHSS of 19 at admission, intravenous thrombolysis, and successful endovascular recanalization of a M2‐MCA occlusion. MRI (3T) at 44 hours after onset revealed a focal hyperperfusion corresponding to the DWI lesion: rCBF = 146%. Patient was discharged with NIHSS = 2 and mRS = 0. (d) Bilateral DWI lesions and asymmetric ASL‐CBF signal. This patient was excluded. A 73‐year‐old‐female patient presenting with left‐sided hemiplegia, dysarthria and neglect, NIHSS of 16 at admission, treated with intravenous thrombolysis. MRI (1.5T) after 53 hours revealed both bilateral ASL and DWI lesions without lateralization. Thus, this patient was excluded from quantitative analyses. He was discharged with NIHSS = 2 and mRS = 1

Patients with favorable and poor outcome at discharge (mRS 0–2 and 3–6, respectively) had also significantly different NIHSS at discharge (0 [IQR 0–2] vs. 2 [IQR 1–4], *p* = 0.020). However, there were no significant differences in initial symptom severity on admission (4 [IQR 1–8] vs. 5 [IQR 2–7], *p* = 0.662). Patients with poor outcome were slightly older (76 vs. 69 years, *p* = 0.083) and more likely to have a relevant vessel pathology (60% vs. 25%, *p* = 0.062; Table 1). Figure 1 displays a selection of representative ASL‐perfusion maps next to the corresponding DWI lesions and a summary of the patients’ clinical presentation.

**Table 1:** Comparison of characteristics for patients with good or poor early outcome

	All patients *n* = 38	Good outcome mRS 0–2 *n* = 28	Poor outcome mRS 3–6 *n* = 10	*p*
Median age (IQR), years	70 (58–77)	69 (58–75)	76 (68–82)	0.083^a^
Female, *n* (%)	15 (39)	12 (43)	3 (30)	0.709^b^
3 Tesla, *n* (%)	22 (58)	18 (64)	4 (40)	0.267^b^
Onset to imaging time, median (IQR), hr	67 (41–109)	76 (45–107)	44 (32–124)	0.334^a^
NIHSS on admission, median (IQR)	4 (1–7)	4 (1–8)	5 (2–7)	0.662^a^
NIHSS at discharge, median (IQR)	1 (0–3)	0 (0–2)	2 (1–4)	0.020^a^
mRS at discharge, median (IQR)	2 (0–3)	1 (0–2)	3 (3–4)	<0.001^a^
Intravenous thrombolysis and/or endovascular recanalization, *n* (%)	11 (29)	9 (32)	2 (20)	0.690^b^
Severe stenosis or occlusion, *n* (%)	13 (34)	7 (25)	6 (60)	0.062^b^
Prior stroke history, *n* (%)	19 (50)	14 (50)	5 (50)	1.000^b^

a Mann–Whitney *U* test, exact, two‐sided *p*‐value given, level of statistical significance set at *p* < 0.05.

b Fisher's exact test; exact, two‐sided *p*‐value given, level of statistical significance set at *p* < 0.05.

### 3.2 Qualitative analysis

Image quality in ASL‐CBF maps and ASL‐BAT maps was comparable in both groups. Most images were rated as being of good or sufficient quality. Asymmetries were detected in 84% of the ASL‐CBF maps and 63% of the ASL‐BAT maps. Twelve out of thirteen patients (92%) with a severe stenosis or occlusion showed an asymmetric ASL‐BAT map and 6/13 (46%) had an ATDA. Patients with good early outcome showed ASL‐BAT asymmetries less frequently (50% vs. 100%, *p* = 0.006; Table 2).

**Table 2:** Comparison of qualitative imaging parameters for patients with good or poor early outcome

	All patients *n* = 38 (%)	Good outcome mRS 0–2 *n* = 28 (%)	Poor outcome mRS 3–6 *n* = 10 (%)	*p*
Quality ASL‐CBF map, *n*				0.167^b^
1 very good	3 (8)	2 (7)	1 (10)	
2 good	27 (71)	22 (79)	5 (50)	
3 sufficient	8 (21)	4 (14)	4 (40)	
Quality ASL‐BAT map, *n*				0.486^b^
1 very good	1 (3)	1 (4)	0	
2 good	22 (58)	18 (64)	4 (40)	
3 sufficient	13 (34)	8 (29)	5 (50)	
4 uninterpretable	2 (5)	1 (4)	1 (10)	
Asymmetry ASL‐CBF, *n*	32 (84)	23 (82)	9 (90)	1.000^a^
Asymmetry ASL‐BAT, *n*	24 (63)	14 (50)	10 (100)	0.006^a^
ATDA, *n*	8 (21)	4 (14)	4 (40)	0.170^a^
Lacunar DWI lesion, *n*	20 (53)	17 (61)	3 (30)	0.144^a^
Infarction in capsula or thalamus, *n*	8 (21)	7 (25)	1 (10)	0.662^a^
DWI lesion in left hemisphere, *n*	23 (61)	18 (64)	5 (50)	0.473^a^
DWI = ASL, *n*	6 (16)	5 (18)	1 (10)	0.885^b^
DWI < ASL, *n*	23 (60)	17 (61)	6 (60)	
DWI > ASL, *n*	9 (24)	6 (21)	3 (30)	

Mann–Whitney *U* test, exact, two‐sided *p*‐value given, level of statistical significance set at *p* < 0.05.

a Fisher's exact test; exact, two‐sided *p*‐value given, level of statistical significance set at *p* < 0.05.

b χ^2^ test; exact, two‐sided *p*‐value given, level of statistical significance set at *p* < 0.05.

While 16% (6/38) of the patients showed ASL perfusion patterns that matched the DWI lesion in extent and location, ASL yielded additive information to DWI in the other 84% (32/38): In 60% (23/38 patients) perfusion anomalies exceeded the DWI lesion and in 24% (9/38 patients) the perfusion alteration was smaller than the DWI lesions or absent.

### 3.3 Quantitative analysis

The quantitative analysis revealed relevant perfusion alterations in 50% of the patients: Hyperperfusion in 7/38 patients (18%) and hypoperfusion in 12/38 patients (32%). The majority of patients with hyperperfusion had a favorable outcome (6/7, 86%), whereas patients with hypoperfusion were distributed equally between both outcome groups (7/12 good outcome, 58%). As expected, a relevant part of patients with an ipsilateral stenosis or occlusion of the ICA or MCA showed hypoperfusion within the stroke lesion (7/13, 54%). There was a trend for worse outcome in patients with a vascular pathology, which did not reach significance (*p* = 0.062). Moreover, patients with vascular pathology showed significantly larger DWI lesions than those without relevant stenosis (8.3 ml [IQR 1.9–27] vs. 0.6 ml [IQR 0.3–2.6]; *p* = 0.008). No such association was found for ASL lesion volumes (62 ml [IQR 16–91] vs. 43 ml [IQR 14.5–60]; *p* = 0.165; Table 3).

**Table 3:** Comparison of quantitative imaging parameters for patients with good or poor early outcome

	All patients *n* = 38	Good outcome mRS 0–2 *n* = 28	Poor outcome mRS 3–6 *n* = 10	*p*
Relevant perfusion alteration, *n* (%)	19/38 (50)	13 (46)	6 (60)	0.714^b^
Hypoperfusion, *n* (%)	12/38 (32)	7/12 (58)	5/12 (42)	0.333^b^
Hyperperfusion, *n* (%)	7/38 (18)	6/7 (86)	1/7 (14)	0.333^b^
Volume of DWI lesion, median (IQR), mL	1.3 (0.4–9.9)	0.6 (0.3–9.5)	5.1 (0.9–17.2)	0.130^a^
Volume of relevant perfusion alteration (*n* = 19), median (IQR), mL	53 (16–65)	53 (14–66)	53 (16–71)	0.765^a^
Volume of hypoperfusion (*n* = 12), median (IQR), mL	63 (51–92)	62 (50–88)	64 (33–95)	1.000^a^
Volume of hyperperfusion (*n* = 7), median (IQR), mL	48 (32–59)	45 (29–61)	48	1.000^a^
Relative CBF (rCBF), median (IQR)	0.95 (0.67–1.15)	1.00 (0.71–1.22)	0.82 (0.44–1.07)	0.177^a^

a Mann–Whitney *U* test, exact, two‐sided *p*‐value given, level of statistical significance set at *p* < 0.05.

b Fisher's exact test; exact, two‐sided* p*‐value given, level of statistical significance set at *p* < 0.05.

### 3.4 Regression model

**Figure 2:**
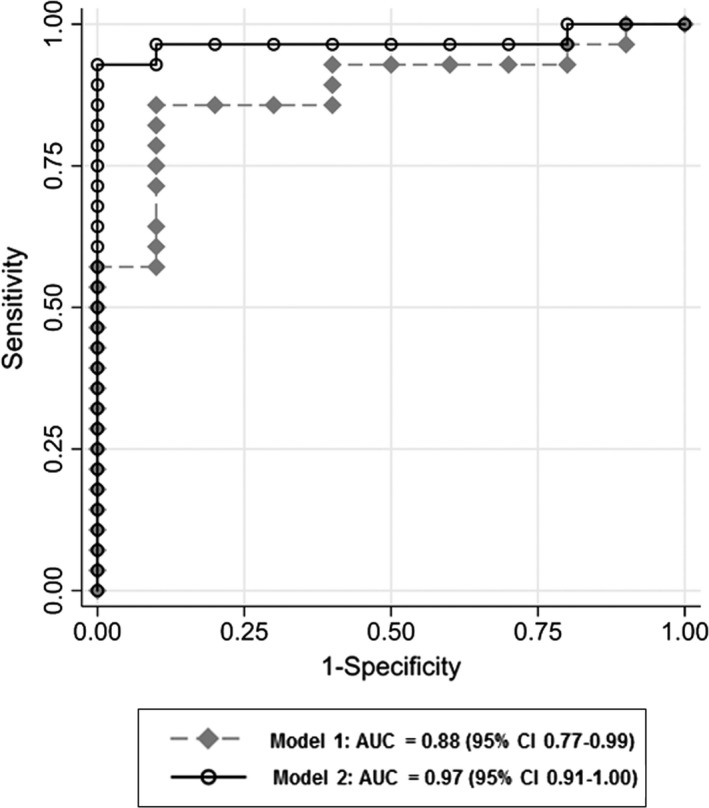
Additional predictive value of arterial spin labeling (ASL) perfusion imaging. Receiver‐operating‐characteristic (ROC) analysis with area‐under‐the‐curve (AUC) and 95% CI of both predictive models: Model 1 (DWI, grey diamonds) and Model 2 (DWI+ASL, open circles). Model 1, which is based on recent clinical standard, is outperformed by Model 2, in which additional ASL perfusion information (hypo‐/hyperperfusion) is added

The major goal of our study was to assess the prognostic benefit of ASL perfusion imaging when added to DWI and clinical data. Consequently, we compared the performance of the two predictive models: Model 1 (DWI) showed an AUC of 0.88 (95% CI 0.77–0.99) with a maximum accuracy of 87%, while Model 2 (DWI + ASL) showed an AUC of 0.97 (95% CI 0.91–1.00) with a maximum accuracy of 95% (Figure 2). Both models were compared using the likelihood‐ratio‐test (*p* = 0.002).

These results demonstrate that when both ASL‐CBF and ASL‐BAT maps are taken into consideration, predictions of early neurological outcome are significantly improved.

## 4 DISCUSSION

ASL perfusion data yield diagnostic and prognostic benefit if added to DWI lesion data and standard clinical parameters of patients with subacute stroke. Moreover, our data indicate that using a commercially available ASL sequence as part of routine stroke imaging protocol provides perfusion data with good diagnostic quality within a short and clinically relevant acquisition time of only 2 minutes.

In line with previous reports, perfusion imaging by ASL detected perfusion alterations with high sensitivity (Zaharchuk et al., 2012) and vessel pathology correlated well with ASL‐BAT map asymmetries (Chng et al., 2008). Our cohort included many small and lacunar infarctions; the majority of these showed absent or minor perfusion alterations, a finding that is in line with previous reports (Kohno et al., 2016). In this study, ASL perfusion alterations were found to be significantly larger than the corresponding infarction as detected by DWI lesion in the majority (84%) of the patients, suggesting that ASL provides additional hemodynamic information beyond structural imaging in those patients.

Previous reports focused on the association of acute stage infarct size, vessel pathology, and clinical data with outcome. Dynamics of cerebral perfusion and tissue at risk in the vulnerable phase following the window of recanalizing therapies are less well understood. However, there is an evidence that the proportion of tissue at risk and infarct core may continue to change for hours or even days and show a large variability between patients (Gonzalez, 2010). We showed that perfusion patterns in subacute stroke, while patients are treated at a stroke unit, can still yield additional relevant information. Our data indicate that at this stage, cerebral perfusion does not merely mirror infarct size or vessel pathology but allows for the detection of perfusion patterns within the ischemic region. Further investigation of associations between specific perfusion patterns and incidence of complications (e.g., intracranial hemorrhage, delirium, and malign media syndrome) in the first days after stroke might lead to better recommendations for vital parameter monitoring, preventive medical treatment, and personalized rehabilitative concepts.

ASL‐based perfusion patterns were able to distinguish between good and poor outcome although initial lesion size and clinical core data were comparable between the groups. The finding that most patients with hyperperfusion (86%) had a good outcome—despite being clinically more affected at baseline—needs further evaluation. It may be linked to endovascular recanalization, as this was a more frequent finding in patients with hyperperfusion (43%) compared to patients with hypoperfusion (17%).

In the predictive modeling, we controlled for potential interactions between ASL perfusion pattern and clinical confounders like endovascular therapy, age, clinical severity, and vascular pathology. The perfusion status was still found to improve prediction regardless of initial therapy.

The significance of ATDA as a marker of hypoperfusion remains controversial. The combination of a delay in the ASL‐BAT map and an ipsilateral reduction of CBF signal associated with hyperintense spots can be interpreted as strong hypoperfusion (Kohno et al., 2016) or, alternatively, as effective leptomeningeal collateralization (Havenon, 2017; Lou et al., 2017). We interpreted ATDA as strong hypoperfusion but new imaging approaches may resolve this artifact in the future (Norris & Schwarzbauer, 1999; Schmid, 2015).

Several limitations must be discussed: First, the sample size is small and heterogenous due to the pilot character of the study. Second, since there was no M0 measurement available, absolute quantification was impossible for the ASL sequence used in this study. To address this issue, relative signal intensity values had to be used and patients with bilateral or complex vessel pathologies, bilateral DWI lesions, or bilateral perfusion alterations had to be excluded. Third, patients in our clinical setting were scanned at two different magnetic field strengths. From a methodological point of view, patients scanned at 1.5T are more likely to show less reliable perfusion values. We addressed this issue by running two repetitions of a multi‐TI imaging scheme. Fourth, prior stroke history may have had an influence on perfusion patterns and recovery, however, there was no evidence of a bias toward changed patient outcome. Fifth, symptom onset to imaging times were broadly heterogeneous and clinical follow‐up was not extended to longer time points. Sixth, our patient population consisted of treated and untreated patients who were admitted to our stroke unit after any form of acute treatment. Hence, differential impact of thrombolysis and thrombectomy prior to imaging cannot be specified in our sample.

The strength of our study is the use of a certified and clinically available sequence with six postlabeling delay time points for brain perfusion assessment and early stroke outcome prediction. Previous reports on association of ASL perfusion imaging and clinical outcome either used single delay ASL (Bivard et al., 2013) or custom‐made ASL sequences (Yu, 2017) in the hyperacute to acute stroke imaging setting (onset to imaging <24 hr). We restricted our study to a clinically approved, multi‐delay imaging protocol that can be implemented into other stroke imaging routines and thus reflects clinical reality.

## 5 CONCLUSION

In subacute stroke, perfusion imaging by a clinically certified ASL sequence provides detailed diagnostic information on regional perfusion dynamics complementary to structural DWI lesion patterns. When added to standard stroke imaging protocols, ASL improves prediction of early neurological outcome in the first week after stroke. This study demonstrates that further clinical validation of ASL and noninvasive perfusion biomarkers in stroke units is merited.

## CONFLICT OF INTEREST

The authors declare no potential conflict of interests.
